# Human-raptor conflict in rural settlements of Colombia

**DOI:** 10.1371/journal.pone.0227704

**Published:** 2020-01-10

**Authors:** Juan Sebastián Restrepo-Cardona, María Ángela Echeverry-Galvis, Diana Lucia Maya, Félix Hernán Vargas, Omar Tapasco, Luis Miguel Renjifo

**Affiliations:** 1 Programa de Maestría en Conservación y Uso de Biodiversidad, Facultad de Estudios Ambientales y Rurales, Pontificia Universidad Javeriana, Bogotá, Colombia; 2 The Peregrine Fund, Boise, ID, United States of America; 3 Departamento de Ecología y Territorio, Facultad de Estudios Ambientales y Rurales, Pontificia Universidad Javeriana, Bogotá, Colombia; 4 Departamento de Matemáticas, Facultad de Ciencias Exactas y Naturales, Universidad de Caldas, Manizales, Colombia; Sichuan University, CHINA

## Abstract

In human-transformed landscapes, predators may feed on domesticated animals, and thus affect human well-being, creating negative perceptions and leading to conflict with people that can result in the persecution of the predator. We studied the factors that influence the perception of the Black-and-chestnut Eagle (*Spizaetus isidori*) in four rural Andean communities in Colombia and compiled historical and recent evidence on the persecution and other causes of mortality of this species in the country. We applied 267 questionnaires and conducted 16 interviews with local inhabitants, via visits to homes and schools in the surroundings of *S*. *isidori* nests. The perception of *S*. *isidori* by the inhabitants was largely negative and influenced by different socio-demographic factors such as gender, chicken (*Gallus gallus*) ownership, and chicken management. The records we obtained indicate that 47 eagles were shot, 16 were captured (three for illegal trafficking) and two were electrocuted on high-tension wires. The persecution of *S*. *isidori* occurs as retaliation or as a preventative measure against chicken predation, and is a significant cause of mortality of this species in Colombia. Effective conservation planning for *S*. *isidori* in Colombia needs to go further than the protected areas system, and include a socioecological perspective in conservation practices applied at landscapes scales that are dominated by people. Education programs and socioecological research, along with participatory work in local communities are key to the conservation of *S*. *isidori* in breeding territories. This approach can also prevent conflict over food resources—*G*. *gallus* and other poultry—that are shared by humans and *S*. *isidori* in rural landscapes.

## Introduction

Tropical deforestation has created a mosaic of landscapes with different degrees of cover and types of use, with some of the original vegetation cover being replaced by anthropic habitats [1,2]. This has increased the frequency of interaction between rural inhabitants and forest species, among them raptors and felines [3,4,5]. These predators usually modify their diet by feeding on domesticated animals, which are more available in anthropized environments [5,6,7], thus affecting human well-being, and creating human-wild life conflict. This conflict can lead to the persecution of the predators, which is particularly worrisome in the case of threatened species whose populations are already decreasing [8,9,10].

Persecution might be the main threat to Neotropical raptors in the form of retaliation, or to prevent predation on domesticated animals [11]. As a consequence, the negative perception of raptors and the resulting actions against them can lead to the extirpation of populations or even to species extinction [12]. The persecution of the Guadalupe Caracara (*Caracara lutosa*) in retaliation for its predation of domesticated animals led to its extinction more than a century ago [12,13,14]. In Ecuador and Brazil, the Harpy Eagle (*Harpia harpyja*) is persecuted to prevent it from preying upon domesticated animals [15,16], even though these animals have not been recorded as part of this eagle’s diet [17,18]. In Argentina, the persecution of the Chaco Eagle (*Harpyhaliaetus coronatus*) results from the attitude of people towards predators in general [19], independent of the fact that domesticated animals make up only 0.2% of its diet [20]. This negative perception and behavior extend to several other predators, such as the Jaguar (*Panthera onca*), for which the perceived impact of domesticated animal loss often exceeds the evidence of attacks [21].

Conflict between humans and predators is defined as the negative interaction between ecological and social elements in a system, which can be studied from different perspectives in both ecological and social research [21,22,23]. In trying to comprehend or attempt to resolve such conflicts, we need to understand how humans perceive the specific wildlife in conflict [24,25] in order to come up with valuable socioecological solutions. Human perception is defined as the human appreciation of biodiversity that influences human behavior towards species [26,27]. Far from being uniform, perception varies among communities [28], differs by gender in some cases [29], and may differ by age [30] and education level [26].

As occurs with other predators that are key to ecosystem processes, the negative perception of raptors results in their persecution, while perpetuating a lack of understanding about their important ecological and cultural roles as ecosystem service providers [31,32]. In order to develop relevant conservation options, research should include the human dimensions, to identify the factors that affect the behavior of people towards these predators [24,27,33].

The Black-and-chestnut Eagle (*Spizaetus isidori*) lives in dense mountain forests throughout the Andes [34]. In Colombia, based on the systematic monitoring of nests, we found that as forest cover decreases in the breeding territories of *S*. *isidori*, the importance of domestic fowl, mainly chickens (*Gallus gallus*), in their diet increases [5]. It is estimated that Colombia’s *S*. *isidori* population includes 160 to 360 pairs, with a worldwide population of less than 1000 adults. This species is therefore classified as Endangered, both nationally and globally [35,36]. The persecution of *S*. *isidori* represents an important threat to this species in the country [36,37,38,39]. In recent years, the original suspicion that the eagle was hunting domestic fowl as part of its diet has been clearly documented [5,40], resulting in its direct persecution.

The aims of this study are to: (1) examine the socio-demographic factors that affect the local inhabitants’ perceptions of *S*. *isidori* in four rural Andean communities in Colombia, and (2) gather historical and recent evidence of the persecution and other causes of mortality of *S*. *isidori* in Colombia. Our first hypothesis is that in sites surrounding nests of this eagle, the perception of this raptor by local inhabitants will be mainly negative and influenced by different socio-demographic factors. Our second hypothesis is that the main cause of *S*. *isidori* persecution is retaliation for or the prevention of chicken predation, and that this is a significant cause of its mortality throughout the country.

## Study area

We surveyed four rural communities in Andean Colombia. The first location in the municipality of Fómeque, Cundinamarca Department, on the eastern slope of the Eastern Andes. Two sites were studied surrounding an *S*. *isidori* nest, La Pastora (04°27'N, 73°50'W) and Mortiñal (04°29'N, 73°53'W), at a mean elevation of 2,600 m a.s.l. The landscape is a mosaic of agricultural crops, open pastures for cattle, human settlements and relicts of Andean riparian, secondary and primary forests (obs. pers.). The second location is in the municipality of Campohermoso, Boyacá Department, on the western slope of the Eastern Andes, where two sites were surveyed at Castañal—Macanalito (05°03'N, 73°08´W) and Huerta Vieja (05°03'N, 73°09'W), at a mean elevation of 2,013 m a.s.l. This landscape is a mosaic of Andean forests, cattle pastures, and heterogeneous agricultural areas, along with shrubby and herbaceous vegetation [5]. The third location, in the municipality of Gigante, Huila Department, on the eastern slope of the Eastern Andes, has a landscape of mixed agricultural crops, open areas for cattle, human settlements and relicts of secondary and primary Andean forest (obs. pers.). The study sites in Gigante were La Umbría (02°15'N, 75°27'W) and Alto de Corozal (02°19'N, 75°28'W), at a mean elevation of 2,096 m a.s.l. The fourth location is in the municipality of Jardín, Antioquia Department, on the eastern slope of the Western Andes. The sites studied around an *S*. *isidori* nest in Jardín were La Floresta–Macanas (05°31’N, 75°52´W) and La Mecenia (05°31’N, 75°51’W), at a mean elevation of 2,036 m a.s.l., where the landscape was also composed of a mix of Andean forest, cattle pastures, heterogeneous agricultural areas and shrubby and herbaceous vegetation [5].

## Materials and methods

### Ethics statement

This study was approved by the Comité de Investigación y Ética from the department of School of Environmental and Rural Studies of the Pontificia Universidad Javeriana, Acta 080.

To examine the perceptions of the rural communities towards *S*. *isidori*, in 2016 and 2017 we interviewed all the people that were found at the time of our visits to homes and schools near the eagle nests in all four localities: 162 men and 105 women over 14 years old. We used this age because in rural Andean communities in Colombia, it is around this age that they are considered young adults [41,42]. The questionnaire had three sections: (1) socio-demographic information about the respondents, including gender, age, level of education, residence location, time of residence, and whether they had ever had any chickens (chicken ownership); (2) characteristics of chicken owners: information on loss of chickens to *S*. *isidori* attacks, and how chickens were kept on the farms; (3) conservation questions related to their willingness to support conservation efforts for the species and, to explore the whether they considered the species an important element in the ecosystem; aiming to explore the inhabitant’s perception of *S*. *isidori*, and their opinion of whether it is a beneficial or harmful species (See S1 Questionnaire).

Additionally, in each locality we conducted semi-structured interviews with two adult women and two adult men to allow them to describe their perception on *S*. *isidori* in greater detail, and used triangulation methodology to analyze the information collected via the questionnaires and the interviews [25]. These people were informed about the aims of the research project and gave their informed consent to participate in the questionnaires and interviews, as recommended by the International Society of Ethnobiology.

To explore non-natural causes of mortality in *S*. *isidori* in Colombia, we compiled information from records of adult and immature eagles being shot at, captured or dying from other causes. This information was obtained by interviewing the ornithological community, and also from wildlife care centers that rescue eagles, the Centro de Rehabilitación de Aves Rapaces—San Isidro (CRARSI) and the Fundación Águilas de Los Andes (FADA). We also included regional public offices in charge of environmental management: the CAM in Huila, CORPOBOYACÁ in Boyacá, CORPOGUAVIO in Cundinamarca, CORPOCESAR, CORPONOR in Norte de Santander, and CORPOURABÁ in Antioquia. Further records were obtained from specimens deposited in the biological collections of the Instituto de Investigación de Recursos Biológicos Alexander von Humboldt (IAvH), the Instituto de Ciencias Naturales de la Universidad Nacional (ICN-UN), the Museo de Ciencias Naturales del Instituto para la Investigación y Preservación del Patrimonio Cultural y Natural del Valle del Cauca (IMCN) and the Natural History Museums of the Universidad de Nariño (MUN) and the Universidad del Cauca (MHN-UC).

### Data analysis

To analyze the inhabitants’ perception of *S*. *isidori*, we carried out binomial tests and Chi-squared tests of independence, with Fisher’s Exact Test when the expected values were less than 5, and the categories were dichotomous [43]. To examine the influence of socio-demographic factors on the perception of *S*. *isidori*, we constructed a binomial logistic model [44]. The explanatory variable was the perception of *S*. *isidori* (beneficial or harmful) and the gender, age, level of education, residence location, time of residence and chicken ownership of the people interviewed were the predictive variables. The sample was divided into two groups, those who had owned chickens in a second explanatory model of the inhabitants’ perception of *S*. *isidori* [26,44]. This was done to analyze the effect of the characteristics associated with owning chickens (having lost chickens to attacks by *S*. *isidori*, and chicken management), including locality and time of residence. Wald’s backward stepwise model was used, with the criterion of significant P values being less than 0.1. All analyses were run in SPSS ^®^ 21. Information regarding the persecution and other causes of mortality for *S*. *isidori* was analyzed descriptively.

## Results

A total of 267 questionnaires were applied. Of those 44.2% were between 27 and 50 years old, and 43.8% were older than 50 years old, followed by those who were between 15 and 26 years old (12%). Regarding education level, 79.8% had finished primary school, 10.9% had received no formal education, and 8.6% had completed secondary school (Table 1).

**Table 1:** Explanatory variables used in the analysis of the perception people have of *Spizaetus isidori* in Colombia, and the frequency of responses by the respondents.

Variable	Type of variable	Response	N	Percent
Gender	Categorical	**•** Female	105	39.3
		**•** Male	162	60.7
Age	Continuous (model)	15–86		
	Categorical (contingency tables)	**•** 15–26	32	12
		**•** 27–50	118	44.2
		**•** > 50	117	43.8
Level of education	Categorical	**•** None	29	10.9
		**•** Primary school	213	79.8
		**•** Junior high school	23	8.6
		**•** High school and higher	2	0.7
Location of residence	Categorical	**•** Gigante	48	18
		**•** Fómeque	69	25.8
		**•** Campohermoso	82	30.7
		**•** Jardín	68	25.5
Time of residence	Continuous (model)	1–86		
	Categorical (contingency tables)	**•** < 15	64	24
		**•** 15–30	83	31.1
		**•** > 30	120	44.9
Chicken ownership	Categorical	**•** Has or has had chickens	228	85.4
		**•** Has never had chickens	39	14.6
Chicken management	Categorical	**•** Not protected^a^	215	80.5
		**•** Protected^b^	52	19.5

^a^Includes people whose chickens are not kept in coops and are therefore not protected from attacks by aerial predators.

^b^Includes those who have chickens in coops and protected from the attacks of aerial predators.

Of the total number of interviewees, 55.4% perceived *S*. *isidori* as a harmful, rather than beneficial bird (44.6%, binomial test, *P* = 0.09). Also, 54.7%, of the respondents said they had not lost any chickens to attacks by *S*. *isidori* (binomial test, *P* = 0.14) and 3.3% acknowledged having killed or knowing someone who had killed an eagle. The majority (77.2%), would be willing to support efforts to conserve the species (binomial test, *P* < 0.001), and 80% feel that *S*. *isidori* is important to the ecosystem (binomial test, *P* < 0.001).

### Effect of the respondents’ socio-demographic characteristics on their perception of *Spizaetus isidori*

**Fig 1:**
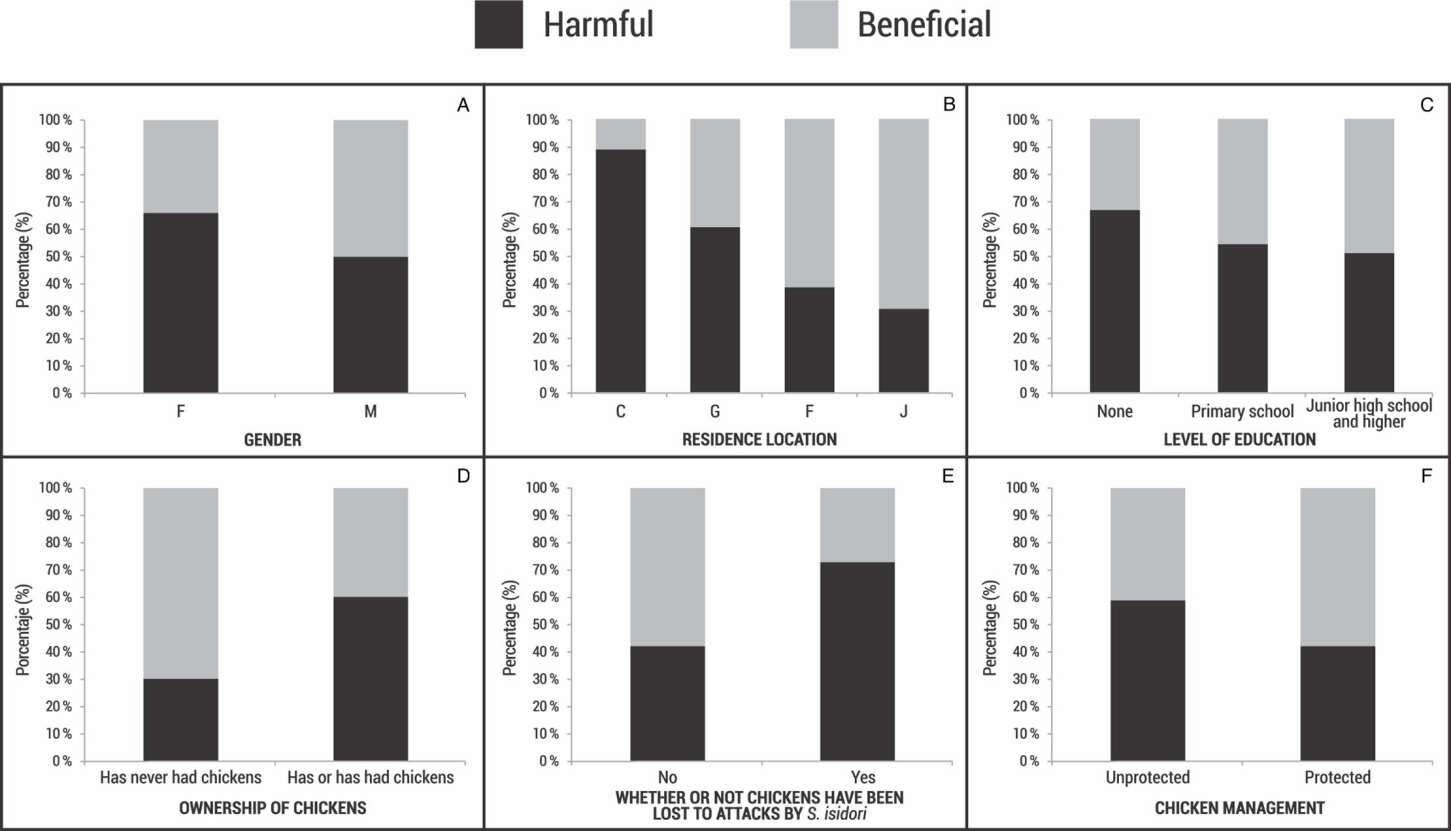
Profiles of the perception of *Spizaetus isidori* by people in Colombia. A) Gender (F: Female; M: Male), B) Residence location (C: Campohermoso; G: Gigante; F: Fómeque; J: Jardín), C) Level of education, D) Ownership of chickens, E) Whether or not chickens have been lost to attacks by *S*. *isidori*, and F) Chicken management (Unprotected: chickens are not kept in coops or protected from aerial attack; Protected: chickens are kept in coops that protect them from aerial predators).

The perception of *S*. *isidori* varied significantly with gender since more women (65.7%) than men (48.8%) had a negative perception of the eagle (X^2^ = 7.4, df = 1; *P* = 0.006; Fig 1A). Similarly, location was a differentiating factor for perception, with those from Campohermoso and Gigante harboring more negative perceptions towards *S*. *isidori* (87.8% and 60.4%, respectively), while in Fómeque (37.7%) and Jardín (30.9%) positive perceptions predominated (X^2^ = 60.6, df = 3; *P* < 0.001; Fig 1B). Perception of this eagle was not related to education level (X^2^ = 1.4, df = 2; *P* = 0.498; Fig 1C), age (X^2^ = 4.8, df = 2; *P* = 0.09) or time of residence (X^2^ = 1.2, df = 2; *P* = 0.53).

### Effect of chicken ownership on the respondents’ perception of *Spizaetus isidori*

The majority of the respondents (85.4%) mentioned having or having had chickens on their farm, with 60.1% expressing a negative perception of the eagle, while of those who had never had chickens, 28.2% perceived the eagle as harmful (X^2^ = 13.7, df = 1; *P* < 0.001; Fig 1D). The negative perception was greater in those who had lost chickens to *S*. *isidori* attacks (X^2^ = 26.8, df = 1; *P* < 0.001; Fig 1E), and varied as chickens were managed in different localities (X^2^ = 4.5, df = 1; *P* = 0.034; Fig 1F). In Campohermoso, Fómeque and Gigante most chicken owners did not keep their chickens in coops that would protect them from aerial predators (92.8%, 92.7% and 89.6%, respectively). In Jardín, 52.9% of the chicken owners kept their fowl in coops.

### Model for analyzing the perception of *Spizaetus isidori* by all the respondents

Based on the binomial logistic model, the factors with a significant impact on the negative perception of *S*. *isidori* (*P* < 0.05) were gender, location of residence and chicken ownership (Table 2). The Hosmer-Lemeshow goodness of fit test shows that the model has an acceptable fit to the data (P = 0.88), as did the cross-classification table (global hit rate of 73.8%), with a Nagelkerke coefficient of determination of 0.35.

**Table 2:** Coefficients of the logistic binomial model for the complete set of interview data to identify which factors are significant in the perception of *Spizaetus isidori* by the inhabitants of Colombia. The variables included in the first step were gender, age, level of education, residence location, time of residence, and chicken ownership.

	B	E.T.	Wald	df	P value	Exp(B)	C.I. 95% for EXP(B)	
							Lower	Upper
**Gender** **Residence location** **Residence location(1)** **Residence location(2)** **Residence location (3)** **Chicken ownership**	0.80	0.30	7.08	1	0.008	2.23	1.24	4.04
			46.66	3	0.000			
	-1.11	0.41	7.41	1	0.006	0.33	0.15	0.73
	1.46	0.46	9.9	1	0.002	4.31	1.73	10.7
	-1.22	0.41	8.67	1	0.003	0.3	0.13	0.67
	1.28	0.43	8.78	1	0.003	0.28	0.12	0.65
**Constant**	0.37	0.34	1.24	1	0.27	1.45		

$$P(Damaging)=\frac{1}{1+exp(-0.37-0.80Sex+1.11Campoh-1.5Fóm+1.22Jard-1.28ChickenOwnershipAC)}$$

B: Coefficient of the variable in the model; E.T. Standard error of the coefficient obtained; Wald: statistic revealing the results of the test of significance for individual parameter; df: degrees of freedom for the test; P value: the probability associated with the statistic obtained, values below the significance level indicate that the variable is significant for the model; Exp. (B) exponential value of the parameter, interpreted as the odds ratio or the ratio of advantage that corresponds to the perception of *S*. *isidori* as harmful, when the person interviewed has a trait of the variable being studied (i.e., gender, residence location, chicken ownership) in comparison to those who do not have the trait of the variable being studied.

### Model for analyzing the perception of *Spizaetus isidori* by those who own chickens

Repeating the binomial logistic model for only those who had chickens, the factors that had a significant impact on the negative perception of *S*. *isidori* (*P* < 0.05) were residence location and having lost chickens to attacks by the eagle (Table 3). The Hosmer-Lemeshow goodness of fit test shows that the model has an acceptable fit to the data (P = 0.83), as did the cross-classification table (global hit rate of 73.7%), with a Nagelkerke coefficient of determination of 0.36.

**Table 3:** Coefficients of the logistic model for evaluating, in the owners of chickens, the characteristics that play a significant role in their perception of *Spizaetus isidori*. Variables included in the first step were residence location, time of residence, whether or not there had been any loss of chickens from attacks by *S*. *isidori*, and chicken management.

	B	E.T.	Wald	df	P value	Exp(B)	C.I. 95% EXP(B)	
							Lower	Upper
**Residence location** **Residence location(1)** **Residence location(2)** **Residence location(3)** **Attacks on chickens** **Constant**			40.72	3	0.000			
	-0.98	0.44	4.86	1	0.03	0.38	0.16	0.90
	1.23	0.52	5.64	1	0.018	3.44	1.24	9.52
	-1.73	0.48	12.88	1	0.000	0.18	0.07	0.46
	-1.20	0.33	12.78	1	0.000	0.30	0.16	0.58
	1.43	0.42	11.73	1	0.001	4.18		

$$P(Damaging)=\frac{1}{1+exp(1.43-0.98Fómeque+1.23Campoh-1.73Garden-1.20ChickenOwnershipAC)}$$

B: Coefficient of the variable in the model; E.T. Standard error of the coefficient obtained; Wald: statistic revealing the results of the test of significance for individual parameter; df: degrees of freedom for the test; P value: the probability associated with the statistic obtained, values below the significance level indicate that the variable is significant for the model; Exp. (B) exponential value of the parameter, interpreted as the odds ratio or the ratio of advantage that corresponds to the perception of *S*. *isidori* as damaging, when the person interviewed has a trait of the variable being studied (i.e., gender, residence location, chicken ownership) in comparison to those who do not have the trait of the variable being studied.

### Persecution and other causes of mortality in *Spizaetus isidori*

We obtained records of 81 *S*. *isidori* killed by different means or captured for a variety of reasons between 1943 and 2019. Of these, 50 were immature and 20 were adults, and for the rest there was no record of age. Forty-seven were killed by gunshot, 16 were captured (three for illegal trafficking), and two were electrocuted on high-tension wires. For the remaining 17 eagles, the cause of mortality was not determined. Of the 63 eagles that were shot or captured, in 60% of the cases the reason given was that the eagles had killed chickens. Only two events (2.5%) were reported between 1943 and 1961, 10% between 1962 and 1980, 12% between 1981 and 1999, and 53% from 2000 to 2019. It was not possible to determine the year when 18 of the deaths occurred.

The 81 reports were for 16 departments: 16 cases for Cundinamarca, 15 cases for Huila, 9 for Boyacá, 6 for Antioquia, 5 for Meta, 4 each for Cauca, Cesar, Putumayo, 3 each for Valle del Cauca, Nariño, Quindío, Magdalena, 2 for Norte de Santander, and one each for Risaralda, Tolima, Santander. For only one event was it not possible to determine the origin of the record (Table 4).

**Table 4:** Records of the hunting, capture, electrocution and illegal trafficking of *Spizaetus isidori* in Colombia, in chronological order.

Date	No. of birds	Sex	Age	Type of incident	Main cause	Department (Location)	Source
Unknown	1	Female	Adult	Unknown	Unknown	Unknown	ICN-UN
Unknown	1	Female	Adult	Capture	Chicken predation	Cesar	CRARSI-FADA
Unknown	1	Unknown	Immature	Gunshot	Unknown	Cundinamarca	IAvH
Unknown	1	Unknown	Unknown	Gunshot	Chicken predation	Antioquia	Alirio Tuberquia
Unknown	1	Unknown	Immature	Gunshot	Chicken predation	Antioquia	Luis F. Quintero
Unknown	1	Unknown	Immature	Gunshot	Unknown	Antioquia	José Castáño
Unknown	1	Unknown	Immature	Gunshot	Unknown	Antioquia	Carlos Restrepo
Unknown	1	Unknown	Unknown	Gunshot	Chicken predation	Putumayo	Alvaro Cardenas
Unknown	1	Unknown	Unknown	Unknown	Chicken predation	Huila	Carlos Fernandez
Unknown	1	Unknown	Adult	Gunshot	Chicken predation	Huila	Edwin Martínez
Unknown	1	Unknown	Adult	Gunshot	Chicken predation	Huila	Edwin Martínez
Unknown	1	Unknown	Immature	Gunshot	Chicken predation	Huila	Edwin Martínez
Unknown	1	Unknown	Unknown	Gunshot	Unknown	Cundinamarca	Santiago Zuluaga
Unknown	1	Unknown	Unknown	Gunshot	Unknown	Cundinamarca	Santiago Zuluaga
Unknown	1	Unknown	Unknown	Gunshot	Unknown	Cundinamarca	Santiago Zuluaga
Unknown	1	Unknown	Unknown	Gunshot	Unknown	Cundinamarca	Santiago Zuluaga
Unknown	1	Unknown	Unknown	Gunshot	Unknown	Cundinamarca	Santiago Zuluaga
Unknown	1	Unknown	Unknown	Gunshot	Unknown	Cundinamarca	Santiago Zuluaga
Aug. 1943	1	Female	Adult	Unknown	Unknown	Cauca	MHN-UC
Jun. 1961	1	Female	Immature	Unknown	Unknown	Cundinamarca	ICN-UN
Aug. 1968	1	Unknown	Immature	Unknown	Unknown	Valle del Cauca	IMCN
1974	1	Unknown	Immature	Unknown	Unknown	Valle del Cauca	IMCN
Feb. 1975	1	Female	Immature	Unknown	Unknown	Cauca	ICN-UN
1976	1	Unknown	Unknown	Gunshot	Unknown	Santander	IAvH
1978	1	Unknown	Immature	Unknown	Unknown	Valle del Cauca	César Márquez
Aug. 1979	1	Male	Immature	Unknown	Unknown	Cauca	MHN-UC
Nov. 1979	1	Female	Immature	Unknown	Unknown	Meta	ICN-UN
1980	1	Unknown	Immature	Unknown	Unknown	Boyacá	César Márquez
1982	1	Unknown	Adult	Gunshot	Unknown	Meta	César Márquez
Jun. 1984	1	Male	Immature	Capture	Illegal trafficking	Nariño	MUN
Dec. 1985	1	Unknown	Adult	Unknown	Unknown	Cundinamarca	ICN-UN
1989	1	Unknown	Unknown	Gunshot	Unknown	Magdalena	IAvH
Aug. 1990	1	Female	Immature	Gunshot	Chicken predation	Nariño	MUN
1995	1	Unknown	Immature	Unknown	Unknown	Boyacá	César Márquez
Feb. 1995	1	Male	Immature	Unknown	Unknown	Boyacá	ICN-UN
1996	1	Unknown	Adult	Capture	Unknown	Nariño	Hector Ramírez
1998	1	Unknown	Adult	Gunshot	Unknown	Huila	CRARSI-FADA
1998	1	Unknown	Adult	Capture	Chicken predation	Boyacá	Santiago Zuluaga
2000	1	Unknown	Adult	Gunshot	Unknown	Norte de Santander	CORPONOR
2000	1	Unknown	Adult	Gunshot	Chicken predation	Boyacá	César Márquez
2002	1	Unknown	Immature	Capture	Unknown	Cundinamarca	CORPOGUAVIO
2002	1	Unknown	Adult	Gunshot	Chicken predation	Cundinamarca	Santiago Zuluaga
Mar. 2002	1	Female	Immature	Gunshot	Unknown	Norte de Santander	IAvH
2003	1	Unknown	Immature	Gunshot	Chicken predation	Boyacá	César Márquez
2004	1	Unknown	Immature	Capture	Illegal trafficking	Cundinamarca	CORPOGUAVIO
2005	1	Unknown	Adult	Gunshot	Chicken predation	Huila	Joaquín Sánchez
2005	1	Unknown	Adult	Gunshot	Chicken predation	Boyacá	César Márquez
2006	1	Unknown	Immature	Unknown	Unknown	Quindío	CRARSI-FADA
Mar. 2006	1	Unknown	Immature	Capture	Chicken predation	Antioquia	CRARSI-FADA
2008	1	Unknown	Immature	Gunshot	Chicken predation	Boyacá	César Márquez
2009	1	Unknown	Immature	Gunshot	Chicken predation	Cundinamarca	CORPOGUAVIO
2009	1	Unknown	Immature	Capture	Chicken predation	Cundinamarca	Carmen Rincón
2010	1	Unknown	Immature	Gunshot	Chicken predation	Cundinamarca	César Márquez
2010	1	Unknown	Immature	Gunshot	Chicken predation	Magdalena	CAR
2011	1	Unknown	Immature	Capture	Unknown	Meta	CRARSI-FADA
2014	1	Unknown	Immature	Gunshot	Chicken predation	Huila	Erik Gaitán
Sep. 2014	1	Unknown	Immature	Accident	Electrocution	Risaralda	CRARSI-FADA
Nov. 2014	1	Unknown	Adult	Capture	Chicken predation	Quindío	CRARSI-FADA
Dec. 2014	1	Unknown	Immature	Gunshot	Chicken predation	Cauca	CRARSI-FADA
2015	1	Unknown	Immature	Gunshot	Chicken predation	Putumayo	Brayan Coral
Jul. 2015	1	Unknown	Immature	Gunshot	Chicken predation	Meta	Iván Sánchez
Nov. 2015	1	Unknown	Immature	Gunshot	Unknown	Huila	CAM
2016	1	Unknown	Adult	Gunshot	Chicken predation	Putumayo	Alvaro Cardenas
Jan. 2016	1	Unknown	Immature	Unknown	Unknown	Quindío	Diana M. Sánchez
Sep. 2016	1	Unknown	Immature	Gunshot	Chicken predation	Huila	CAM
Oct. 2016	1	Unknown	Immature	Capture	Illegal trafficking	Meta	CRARSI-FADA
2017	1	Unknown	Immature	Gunshot	Chicken predation	Huila	Edwin Martínez
Feb. 2017	1	Unknown	Adult	Gunshot	Unknown	Boyacá	CORPOBOYACÁ
Jun. 2017	1	Unknown	Immature	Gunshot	Chicken predation	Putumayo	Alvaro Cardenas
Aug. 2017	1	Unknown	Immature	Capture	Chicken predation	Huila	CAM
Oct. 2017	1	Female	Immature	Capture	Chicken predation	Cesar	CORPOCESAR
Feb. 2018	1	Female	Adult	Gunshot	Unknown	Antioquia	CORPOURABÁ
Apr. 2018	1	Male	Immature	Gunshot	Chicken predation	Cesar	CORPOCESAR
Sep. 2018	1	Unknown	Immature	Gunshot	Chicken predation	Cesar	CRARSI-FADA
Oct. 2018	1	Male	Immature	Gunshot	Unknown	Huila	CAM
Apr. 2019	1	Unknown	Immature	Capture	Chicken predation	Magdalena	Tony Cala
May. 2019	1	Unknown	Adult	Gunshot	Unknown	Huila	IAvH
Jun. 2019	1	Unknown	Immature	Gunshot	Chicken predation	Tolima	IAvH
Jul. 2019	2	Unknown	Immature	Capture	Chicken predation	Huila	CAM
Oct. 2019	1	Unknown	Immature	Accident	Electrocution	Cundinamarca	ICN-UN

## Discussion

The perception of *S*. *isidori* was mostly negative and influenced by different socio-demographic factors such as gender, residence location, chicken ownership, having lost chickens to predation by *S*. *isidori* and the way chickens were managed on farms (Fig 1). The persecution of this eagle occurs as retaliation or as a preventative measure against chicken predation, and is a significant cause of mortality of this species in Colombia, which is also threatened by the possibility of electrocution and illegal trafficking (Table 4); though it is unknown to what extent the latter affect *S*. *isidori* populations in the country. Additionally, there is the effect of habitat loss; the species has lost 60% of its original habitat [35].

Men and women in rural communities, have different roles in the agricultural practices, management and appreciation of natural resources, resulting in different perceptions and behaviors towards wildlife [26,29,45]. At the four study sites, women had a more negative perception of *S*. *isidori* (Fig 1), probably because in rural Andean communities in Colombia, the women take care of household and animals such as chickens [41,42]. For conservation actions based on the perceptions of local communities, it is important to identify this type of gender difference and its relationship to the use and management of natural resources [46,47,48]. This is why—even though in biodiversity conservation programs the gender of the respondents is considered secondary or a distraction [49]—our results highlight the relevance of taking gender into account in social research and education programs to protect *S*. *isidori* populations; this information could lead to different strategies for involving men and women in conservation efforts.

The way in which chickens were managed also affected people’s perception of *S*. *isidori*, and was related to chicken ownership and location (Fig 1). Of the four locations, in Campohermoso and Gigante, *S*. *isidori* was negatively perceived. In Campohermoso, a location with less forest cover, and where most of those who owned chickens said they did not keep their fowl inside coops or protected from aerial predators, *S*. *isidori* fed mainly on the chickens. Meanwhile, in Jardín, where forest cover is about 66% more abundant and where most of the chicken owners do have coops, *S*. *isidori* preyed mostly on arboreal mammals and consumed relatively fewer chickens [5]. These findings suggest that the variation in chicken consumption by *S*. *isidori*, i.e. something that affects how the eagle is perceived, might be related to both deforestation and management of domestic fowl [5,10,50]. Thus, in locations where there is conflict between the local communities and *S*. *isidori*, it is important to develop various approaches to conservation. These evidence-based management actions for mitigating conflict between people and *S*. *isidori* could include maintaining or even increasing forest cover, enhancing populations of the eagle´s arboreal mammal prey species, reducing the exposure of chickens by using enclosures, and offering economic compensation when chickens are eaten by this eagle.

Prior experience of predation on their fowl, clearly influenced people’s perception towards wildlife as was recorded in this study (Fig 1). Interactions between people and predators result in the adoption of a certain perception of these animals, affecting how people behave towards them [21,30]. The persecution of *S*. *isidori* is, in fact, a significant cause of mortality that occurs across much of the country (16 departments), making it a national conservation issue since 60% of the cases of persecution occurred as retaliation or in an effort to prevent the predation of chickens (Table 4).

Most of the respondents said they would be willing to support the conservation of *S*. *isidori* (77.2% of all those interviewed), and 81% recognized the importance of this species to the ecosystem. People often mentioned that *S*. *isidori* feeds on animals that can enter homes and cause damage, such as snakes and rodents. Greater knowledge of the ecology of this eagle and associated ecosystem services on the part of the local inhabitants can lead to public support for conservation strategies [27,33]. Even though the level of education was not a factor that significantly affected the perceptions of people towards *S*. *isidori*, the people who mentioned having no formal education were those who mostly had a negative perception of the species (Fig 1). This suggests that, environmental education would be useful for increasing public awareness of the raptors that are endangered, and would promote positive behavior towards these predators [51]. This is supported by the fact that in Argentina, rural inhabitants with higher levels of education tended to have a more positive perception of the Andean Condor (*Vultur gryphus*) [26]. In the breeding territories of *S*. *isidori* in Colombia, it is important to develop programs that increase public knowledge of this species and clearly state its benefits to the ecosystem. A special effort should be made to include women, those who own chickens and those who said they have killed eagles, as they are most likely to have a negative perception of *S*. *isidori*.

The protection of large raptors often requires the preservation of extensive natural areas where there has been little anthropic disturbance [52,53]. However, human-transformed habitats such as the rural Andean landscapes of Colombia [54,55] can provide habitat for the species that may tolerate some degree of anthropic disturbances [4,5]. Effective conservation planning for *S*. *isidori* in Colombia needs to go further than the protected areas system, which has been the historical approach [35, 56], and include a socioecological perspective in conservation practices applied to landscapes dominated by people. Education programs and socioecological research, along with participatory work in local communities are key to the conservation of *S*. *isidori* in breeding territories. This would prevent competition for food resources and subsequent conflict in rural landscapes where raising chickens is a common practice (85.4% of all those interviewed), and where they represent a relatively important food source for the Black-and-chestnut Eagle [5].

## Supporting information

S1 Questionnaire Questionnaire to examine the perceptions of the rural communities towards the Black-and-chestnut Eagle (*Spizaetus isidori*). The questionnaire had three sections: (1) socio-demographic information about the respondents; (2) characteristics of chicken owners; (3) conservation questions to explore the inhabitant’s perception of *S*. *isidori*.
(PDF)

S1 Dataset All data underlying the findings reported. (XLSX)
