# Supplementary material for: Human-raptor conflict in rural settlements of Colombia
Source: PLoS One. 2020 Jan 10;15(1):e0227704. doi: 10.1371/journal.pone.0227704 (PMC6953857; doi:10.1371/journal.pone.0227704)
Supplement: S1 Questionnaire — The questionnaire had three sections: (1) socio-demographic information about the respondents; (2) characteristics of chicken owners; (3) conservation questions to explore the inhabitant’s perception of S. isidori. (PDF) [file pone.0227704.s001.pdf]

S1 Questionnaire. Questionnaire to examine the perceptions of the rural communities towards the Black-and-chestnut Eagle (*Spizaetus isidori*). The questionnaire had three sections: (1) socio-demographic information about the respondents; (2) characteristics of chicken owners; (3) conservation questions to explore the inhabitant's perception of *S. isidori*.

Questionnaire No.____________________ Date: ____________________

Questionnaire – Perceptions towards the Black-and-chestnut Eagle

1. Name: ____

2. Gender: a. Male ( ) b. Female ( )

3. Age: a. 10-14 years ( ) b. 15-26 years ( ) c. 27-30 years ( ) d. 31-39 years ( )
e. 40-50 years ( ) f. 51-60 years ( ) g. + 60 years ( )

4. Level of education:
a. Primary school ( ) b. Junior high school ( ) c. High school and higher ( ) d. none ( )

5. Address:
Site ________________ Municipality______________Department _________________

6. Indicate the time you have been living in this region:
a. Less than 1-year ( ) b. Between 1-5 years ( ) c. Between 6-15 years ( )
d. Between 15-30 years ( ) e. More than 30 years ( )

7. Do you keep, or have you kept chickens on your farm?
If the answer is Yes, please continue to question 8
If the answer is No, please continue to question 10

8. Do you keep chickens in coops or protected from aerial attack?
If yes ( ) If not ( )

9. Has the Black-and-chestnut Eagle attacked chickens on its farm?
If yes ( ) If not ( )

10. Is the Black-and-chestnut Eagle important for the ecosystem?
If yes ( ) If not ( )
Why?
____
____
____

11. Would you be willing to support the protection of the Black-and-chestnut Eagle on your farm?
If yes ( ) If not ( )
Why?

12. Have you killed a Black-and-chestnut Eagle?
If yes ( ) If not ( )
If the answer is Yes, please describe when, how, where and why:

13. Consider that the Black-and-chestnut Eagle is: a. Beneficial ( ) b. Harmful ( )
